# Knuckle Pigmentation as an Early Cutaneous Sign of Vitamin B12 Deficiency: A Case Report

**DOI:** 10.31729/jnma.5048

**Published:** 2020-10-31

**Authors:** Ankita Srivastava, Sanjiv Choudhary

**Affiliations:** 1 Department of Dermatology, All India Institute of Medical Sciences, Nagpur, Maharashtra, India

**Keywords:** *knuckle pigmentation*, *treatment*, *vitamin B12 deficiency*

## Abstract

Vitamin B12 deficiency can present with variable hematological, neuropsychiatric, and mucocutaneous changes. Hyperpigmentation, specifically involving the knuckles has been described in vitamin B12 deficiency, but usually,these patients are symptomatic with systemic manifestations like megaloblastic anemia, pancytopenia, or neurological deficits. Here, we are reporting a case of nutritional vitamin B12 deficiency, who presented with isolated knuckle pigmentation and was successfully treated with oral therapy. This case also highlights the importance of recognizing this cutaneous sign as an early marker of vitamin B12 deficiency; thereby enabling the clinician to treat the disease before it leads to irreversible neurological complications.

## INTRODUCTION

Pigmentary changes have been described in several cases of vitamin B12 deficiency.^1^ These changes often occur in association with systemic features such as megaloblastic anemia, pancytopenia, and neurological deficits.^2^ Here, we report a case of vitamin B12 deficiency who presented to the dermatologist with the sole complaint of hyperpigmentation over knuckles. She was not found to have any associated systemic abnormality; thereby highlighting the importance of this cutaneous sign in early detection and treatment of this deficiency.

## CASE REPORT

**Figure 1:**
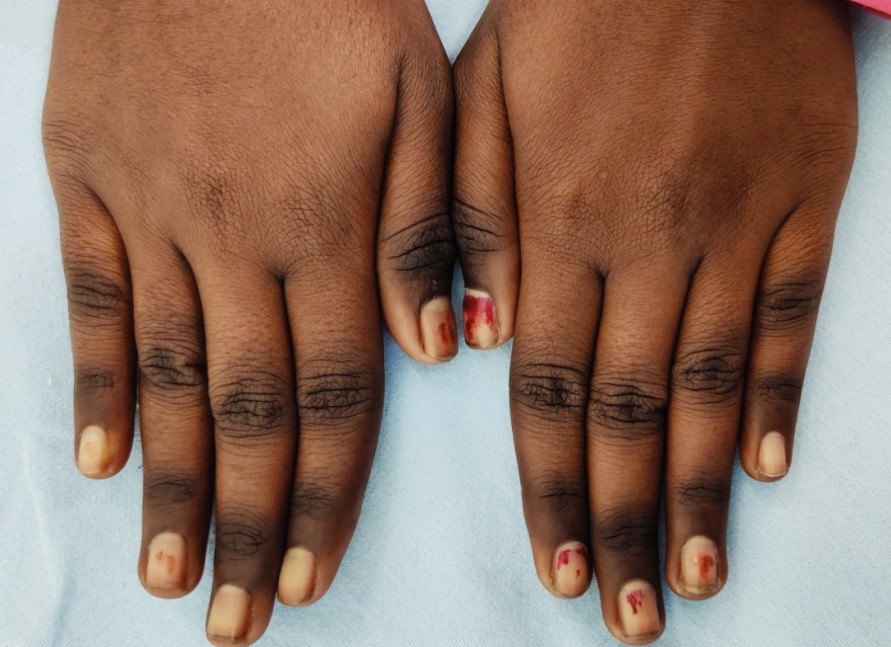
Localized hyperpigmentation over the knuckles.

A 29-year old female presented to the dermatology clinic with complaints of pigmentation over the dorsa of both hands for the past two months. There was no history of any preceding eruption, drug intake, topical application, or excessive sun exposure. She had been on a strictly vegetarian diet since childhood. There was no history of diarrhoea, weight loss, or other gastrointestinal complaints. On examination, well-demarcated areas of pigmentation localized to knuckles of both hands were noticed (Figure 1).

Neurological examination was within normal limits. On laboratory evaluation, serum vitamin B12 level was 192 pg/ml (reference range: 239-931), Hemoglobin was 13.4 g per deciliter, total leucocyte count was 4.5 × 10^9^ per liter and platelet count was 317 × 10^9^ per liter. The mean corpuscular volume was 86.4 fl. Peripheral blood film revealed a normocytic, normocytic picture with a normal appearance of leucocytes and an adequate number of platelets.

Based on relevant history, clinical and laboratory findings, the diagnosis of nutritional vitamin B12 deficiency was established and the patient was treated with oral vitamin B12 in a dose of 1500 μg per day. After 2 months, the serum vitamin B12 level improved to 577 pg/ml and the pigmentation began to regress. Considering the dietary habit, she has been advised to continue with oral supplementation of vitamin B12 in a dose of 5 μg daily along with iron and folic acid.

## DISCUSSION

The deficiency of vitamin B12 often manifests as hematological and neurological findings. Pigmentary changes in the form of pigmentation of knuckles, oral mucosa, and Addisonian pigmentation have also been described.^1^ However, most of these cases presented with systemic manifestations like anemia, pancytopenia, malabsorption, and variable neuropsychiatric disorders.^1–3^ Our case, however, presented with isolated knuckle pigmentation, and no systemic changes were found on detailed evaluation.

The deficiency of vitamin B12 is defined as a plasma vitamin B12 level of less than 200 pg/ml.^1^ In the present case, since vitamin B12 level was slightly below the cut-off, it can be labeled as a relatively mild or early stage of deficiency, due to which no systemic changes had developed. But, if it is not detected and treated at this stage, it can inevitably progress to hematological and neurological complications; some of which can lead to permanent disability.^4^

We could not order for antibodies directed against parietal cells and intrinsic factor in view of poor socio-economic background of the patient. Also, since she had a strict vegetarian diet and had no gastrointestinal complaints, it was more likely to be due to nutritional deficiency rather than malabsorption. The diagnosis was further supported by excellent clinical response to oral vitamin B12.

We chose oral therapy for the patient as there was no neurological involvement. It also helped to improve patient compliance as it was convenient for her to take treatment at home and avoided the loss of daily wages on account of coming to the hospital for injections. It has been demonstrated in several studies that oral supplementation is as effective as intramuscular injections, and oral therapy can be utilized even in cases of pernicious anemia.^5^ It also reduces the cost of therapy.^6^ However, it must be noted that patients with severe neurological deficits or critically low levels of vitamin B12 should be treated with parenteral therapy, to ensure rapid replenishment of body stores to prevent irreversible consequences. Such patients can later be shifted to oral therapy.^5^

The majority of the Indian population is at high risk of developing nutritional vitamin B12 deficiency, due to strict vegetarian food habits. Knuckle pigmentation is an easily visible and early sign of vitamin B12 deficiency. It may occur even before the development of hematological and neurological complications. Therefore, clinicians especially dermatologists must be aware of this sign so that such cases may be diagnosed and treated early. Further, these cases can be treated successfully with oral therapy, which reduces the cost and discomfort associated with injections.

## Consent:

**JNMA Case Report Consent Form** was signed by the patient and the original article is attached with the patient's chart.

## Conflict of Interest

**None.**
